# Effect of hydroxychloroquine use on the length of hospital stay in children diagnosed with COVID-19

**DOI:** 10.14744/nci.2021.65471

**Published:** 2022-01-03

**Authors:** Hatice Uygun, Mehmet Turgut, Habip Almis, Ibrahim Hakan Bucak

**Affiliations:** 1. Department of Pediatric Infectious Disease, Adiyaman University Faculty of Medicine, Adiyaman, Turkey; 2. Department of Pediatrics, Adiyaman University Faculty of Medicine, Adiyaman, Turkey

**Keywords:** Children, coronavirus, apidemiological data, hydroxychloroquine, pandemics

## Abstract

**Objective:**

COVID-19 since the reporting of the first case of infection and its declaration as a pandemic, it caused morbidity and mortality in hundreds of thousands of people. In the early stages of the COVID-19 pandemic, the number of confirmed cases among children was relatively low, and therefore, data were limited. However, the number of pediatric cases has also risen markedly among children in the later stages of the pandemic.

**Methods:**

Forty patients from 1 month to 18 years of age who presented to the Division of Pediatric Infectious Diseases of a tertiary research and training hospital between March 10, 2020, and May 31, 2020, with symptoms suggestive of COVID-19 infection and whose combined oropharyngeal and nasopharyngeal swab specimens tested positive on real-time reverse transcription polymerase chain reaction (rRT-PCR) were included in the study.

**Results:**

Forty pediatric patients with a mean age of 109.1±66.1 months were included in the study. Among patients, 62.5% (25/40) were girls and 37.5% (15/40) were boys. The most presentation symptom was cough in 19 (47.5%) patients. Hydroxychloroquine (HQ) therapy was given as part of combination treatment to 15 symptomatic patients older than 72 months of age (72–143 months of age: 4 patient, 144–216 months of age: 11 patients). The mean time to a rRT-PCR negative test was 7.2±1.69 (4–10) days for the group receiving an HQ treatment protocol and 8.2±1.44 (6–11) days for the group receiving a non-HQ treatment protocol with no significant difference between the groups (p=0.054).

**Conclusion:**

In this study, it was shown that the use of HQ had no effect on the length of hospital stay and that there was no significant difference between patients in terms of epidemiological data.

**C**oronaviruses are RNA viruses from the Coronaviridae family. These viruses cause infection in most mammals including humans [1]. While human coronavirus infections are mostly self-limiting with a mild course, severe acute respiratory syndrome coronaviruses (SARS-CoV) and the Middle East respiratory syndrome coronavirus (MERS-CoV) posed a serious threat within the past 20 years with a mortality rate of 10% and 37%, respectively [2, 3]. A novel strain of coronavirus appeared in December 2019 in the city of Wuhan in Hubei Province, China and spread quickly worldwide. This novel coronavirus was previously referred to as 2019 novel coronavirus (2019-nCoV) and subsequently named as SARS-CoV-2. COVID-19 is the name given by the WHO for the disease caused by the novel coronavirus SARS-CoV-2. This novel microorganism was identified through research efforts prompted by the observation that affected patients had similar pneumonia symptoms and Jiatong et al. [4] demonstrated that as with SARS-CoV, SARS-CoV-2 also enters the cell by binding to the angiotensin converting enzyme 2 (ACE-2) cell receptor [5]. Currently, the COVID-19 represents a significant global threat since SARS-CoV-2 is a more infectious virus than other major members of the coronavirus family such as those causing SARS and MERS and transmitted rapidly among humans. The WHO announced COVID-19 outbreak as a pandemic on March 11, 2020. Since the reporting of the first case of infection and its declaration as a pandemic, COVID-19 caused morbidity and mortality in hundreds of thousands of people [6, 7].

In the early stages of the COVID-19 pandemic, the number of confirmed cases among children were relatively low, and thus, data were limited. However, the number of pediatric cases has also increased markedly to date among children. Many authors have emphasized that especially children with comorbid conditions should be considered as a high-risk group [8].

This study aimed to evaluate epidemiological, clinical, laboratory, and radiological findings and treatment outcomes of patients who were diagnosed with 2019-nCoV infection and received in-hospital treatment at the Division of Pediatric Infectious Diseases of a tertiary research and training hospital.

## Materials and Methods

### Subjects

**Figure 1.**
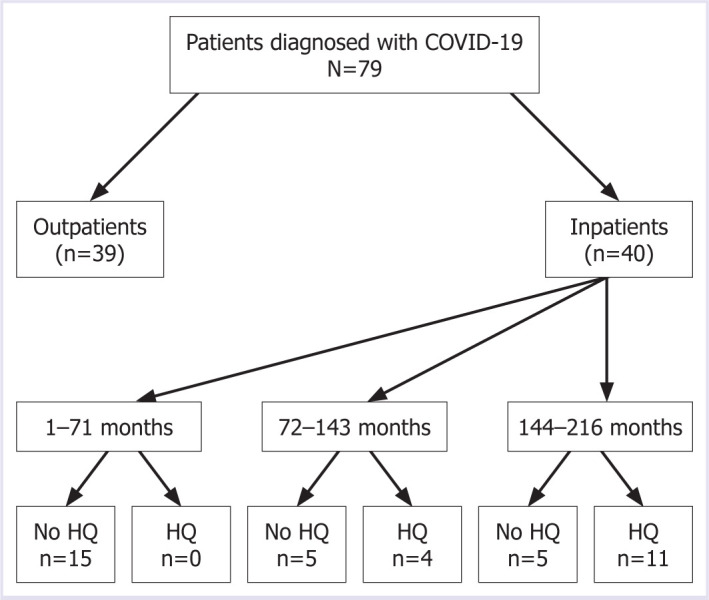
Classification of patients as per study inclusion criteria (age-treatment).

Forty patients from 1 month to 18 years of age who presented to the Division of Pediatric Infectious Diseases of a tertiary research and training hospital between March 10, 2020, and May 31, 2020, with symptoms suggestive of COVID-19 infection (history of contact with adults diagnosed with COVID-19, fever, cough, tachypnea, respiratory distress, loss of taste sensation, and loss of sense of smell) and whose combined oropharyngeal and nasopharyngeal swab specimens tested positive on real-time reverse transcription-polymerase chain reaction (rRT-PCR) were included in the study. All patients were diagnosed with SARS-CoV-2 infection and subsequently hospitalized and monitored due to their symptoms (Fig. 1). The study patients were divided into three age groups (Groups 1–3). Laboratory parameters, radiological findings, length of hospital stay, and response to treatment were reviewed and compared among these groups. The patients were also divided into six subgroups based on the treatment they received:

**Highlight key points**

• Currently, there is no effective treatment method for COVID-19 infection in children.
• It was determined that the use of HQ in pediatric cases diagnosed with COVID-19 did not affect the length of hospital stay.
• In the light of the data obtained in the study, it was determined that the age of the patient was not one of the parameters affecting the length of hospital stay.

The study groups were as follows:

• Group A: Children from 1 to 71 months age receiving a treatment protocol with hydroxychloroquine (HQ)
• Group B: Children from 1 to 71 months age receiving a treatment protocol without HQ
• Group C: Children from 72 to 143 months age receiving a treatment protocol with HQ
• Group D: Children from 72 to 143 months age receiving a treatment protocol without HQ
• Group E: Children from 144 to 216 months age receiving a treatment protocol with HQ
• Group F: Children from 144 to 216 months age receiving a treatment protocol without HQ.

All patients had prior contact with adults with COVID-19 infection and none had a history of travel to foreign countries.

Clinical classification of the study patients in terms of pneumonia was performed according to the COVID-19 (SARS CoV-2 infection) guidelines issued by Republic of Turkey Ministry of Health General Directorate of Public Health updated on June 1, 2020. The guidelines state that uncomplicated group consists of patients without an underlying illness who have normal findings on posterior-anterior chest radiographs or thorax computed tomography (CT) and non-specific findings including fever, malaise, mild cough, and sore throat. According to the guidelines, to be considered as having pneumonia, the patients should have characteristic imaging findings specific to COVID-19 on chest X-rays or CT scans, but respiration rate should fall within normal range for age and/or oxygen saturation at room temperature should be 90% or greater. Severe/very severe pneumonia group consists of patients with tachypnea and/or low oxygen saturation who have dense, typical infiltrations detected on thorax CT scans, require mechanical ventilation, developed acute organ dysfunction or are in a state of shock [9]. In our study, none of our patients had severe pneumonia findings. For this reason, the patients were divided into two main groups, different from the COVID-19 (SARS CoV-2 infection) guideline published by the Ministry of Health General Directorate of Public Health, which was updated on June 1, 2020. They were categorized as patients with uncomplicated disease and those who met criteria for pneumonia and/or severe pneumonia based on their clinical and radiological findings. Although none of the patients had a concurrent chronic condition, all were followed as in-hospital patients because they had a high number of household contacts and were symptomatic. HQ was not given to any patient younger than 72 months as it was not recommended in the guidelines. However, due to the lack of consensus regarding the treatment of COVID-19 infection in children, HQ treatment was given to patients with signs and/or symptoms of pneumonia and those who did not have pneumonia but had severe symptoms and/or signs. Treatment response was adjudicated on the basis of improvement of blood parameters and clinical course and becoming negative on rRT-PCR test. All patients were discharged on becoming rRT-PCR test-negative.

### Study Data

In this retrospective study, age, sex, presence of an underlying illness, history of exposure to SARS CoV-2, presenting symptoms, physical examination findings, radiological imaging results (posterior-anterior chest radiographs and thorax CT), laboratory findings, treatments administered, clinical course, time from admission to becoming negative on rRT-PCR test (days), and length of hospital stay (days) of the patients were retrieved from electronic files.

For laboratory data, complete blood count parameters white blood cell (WBC) count, Hemoglobin (Hb) level, lymphocyte, neutrophil, platelet (PLT) counts were recorded. Serum biochemistry included lactate dehydrogenase, aspartate aminotransferase (AST), alanine aminotransferase, total bilirubin, creatine kinase (CK), creatinine, urea, alkaline phosphatase (ALP), and gamma-glutamyl transferase. In addition, Troponin I, CK myocardial band, D-dimer, C-reactive protein, procalcitonin, Vitamin B12, folate and prothrombin time, activated partial thromboplastin time (Aptt), international normalized ratio levels were recorded. For differential diagnosis, influenza A virus immunoglobulin (Ig) M, influenza B virus Ig M, adenovirus Ig M, parainfluenza virus Ig M, respiratory syncytial virus Ig M, Mycoplasma pneumonia Ig M, Chlamydia pneumonia Ig M and legionella pneumonia Ig M tests were performed to exclude other infectious agents.

### Statistical Analysis

SPSS version 22 for Windows (SPSS, Chicago, IL, USA) was used for statistical analysis. Descriptive statistics were used to analyze the study data. All data were presented as mean±standard deviation and percentage. Intra-group comparisons were done using the Student’s t-test for parametric data. Non-parametric data were compared using the Chi-square test.

Ethics approval for the conduct of the study was obtained from the Institutional Review Board (Approval No: 2020/6-53) as well from Turkish Ministry of Health (Approval No:2020-05-13T12_02_01).

## Results

Forty pediatric patients with a mean age of 109.1±66.1 months were included in the study. Among patients, 62.5% (25/40) were girls and 37.5% (15/40) were boys. The patients were divided into three main groups based on age: 1–71 months (Group 1, mean age 25.2±21.2 [2–67] months), 72–143 months (Group 2, mean age 114.3±25.7 [75–142] months), and 144–216 months (Group 3, mean age 175.3±21.5 [145–213] months).

The presentation symptoms were high-grade fever in 8 (20%) patients, cough in 19 (47.5%) patients, weakness in 14 (35%) patients, loss of taste sensation in 2 (5%) patients, loss of sense of smell in 2 (5%) patients, diarrhea in 1 (2.5%) patients, and vomiting in 1 (2.5%) patient (Table 1).

**Table 1.** Presenting symptoms

Symptom	Total (%)	Boys (%)	Girls (%)
Fever	20	10	10
Cough	47.5	22.5	25
Weakness	35	7.5	27.5
Diarrhea	2.5	2.5	0
Vomiting	2.5	2.5	0
Loss of taste sensation	5	0	5
Loss of sense of smell	5	0	5
Total	47	18	29

Posterior-anterior chest radiographs were obtained for all patients, six of whom did not show infiltration. Posterior-anterior chest radiographs showed dense infiltration bilaterally in five patients and paracardiac infiltration in 15 patients and chest radiography findings consistent with viral pneumonia in 14 patients. Of these patients, 17 underwent thorax CT imaging which showed areas of ground glass opacity in three patients. Thorax CT findings of other patients were normal. Pneumonia classification was done based on radiological findings and only 3 (8.1%) patients who met the criteria for pneumonia were monitored. Clinical condition of the patients did not worsen during follow-up. None of the patients required intensive care and mechanical ventilation. Other three patients were excluded from pneumonia classification because they were diagnosed with COVID-19 due to extra-pulmonary signs and symptoms.

Laboratory parameters of the study patients are presented in Table 2. Associations between mean laboratory values and age groups were analyzed. Statistical analyses showed significant differences among age groups with regard to the mean values of WBC, Hb, lymphocyte count, PLT count, AST, ALP, Aptt, vitamin B12, folate, (p<0.05). When the results of the groups were evaluated, the change in laboratory parameters compatible with COVID-19 infection was more pronounced in the older age group. In the statistical evaluation, a significant difference was observed in WBC, Hb, and PLT values between Groups 1 and 3 (p=0.00, p=0.00, p=0.032). The decrease in lymphocyte count became more pronounced with increasing age, and a statistically significant difference was observed between the groups (p=0.00). Vitamin B12 and folate levels were measured lower with increasing age, and a statistically significant difference was observed between Groups 1 and 3 in the evaluation (p=0.022), (p=0.026). In addition, a statistically significant difference was found between Groups 1, 2, and 3 for AST value (p=0.00), for ALP value between Groups 2 and 3 (p=0.011), and for Aptt value between Groups 1 and 3 (p=0.003).

**Table 2.** Laboratory parameters by age group

	Group-1 Mean±SD (Min.–Max.) n=15	Group-2 Mean±SD (Min.–Max.) n=9	Group-3 Mean±SD (Min.–Max.) n=16	Total Mean±SD (Min.–Max.) n=40	p
UREA (mg/dL)	23.1±10.01 (8–47)	22.8±5.5 (13–30)	21.6±5.2 (13–31)	22.5±7.3 (8–47)	0.935
ALT (U/L)	24.8±13.9 (14–59)	19.0±7.8 (11–35)	17.4±6.3 (9–31)	20.5±10.4 (9–59)	0.350
AST (U/L)	36.4±14.7 (21–79)	25.1±5.4 (18–36)	18.2±5.5 (13–33)	26.6±12.7 (13–79)	0.001
ALP (U/L)	190.3±41.9 (125–282)	253.1±59.8 (179–350)	154.8±91.1 (53–396)	188.5±78.6 (53–396)	0.009 Aptt (sec)	27.1±4.8 (16.9–34.2)	28.6±2.8 (21.7–30.8)	31.8±2.3 (26.9–35.6)	29.4±4.0 (16.9–35.6)	0.011
INR (s)	1.00±0.11 (0.87–1.29)	1.02±0.08 (0.90–1.18)	1.15±0.42 (0.90–2.72)	1.06±0.28 (0.87–2.72)	0.514
LDH (U/L)	262.3±61.2 (157–336)	254.4±116.3 (154–546)	119.5±81.5 (116–350)	233.4±88.9 (116–546)	0.238
WBC (10^3^/uL)	8.63±2.58 (3.9–13.0)	6.71±2.19 (4.1–10.8)	5.18±1.42 (3.1–8.0)	6.82±2.55 (3.1–13.0)	0.00
Lymphocyte count (10^3^/uL)	4.95±2.54 (1.3–10.7)	2.48±7.20 (1.2–3.5)	1.73±0.423 (1.1–2.6)	3.11±2.16 (1.1–10.7)	0.00
Hb (g/dL)	12.0±1.3 (10–15)	13.1±0.7 (12–14)	13.1±1.1 (11–15)	12.7±1.2 (10–15)	0.083
Neutrophil count (10^6^/uL)	2.5±1.7 (0.5–6.8)	3.1±1.6 (1.0–5.1)	2.86±1.4 (0.9–6)	2.8±1.5 (0.5–6.8)	0.261
PLT count (10^3^/uL)	324.7±120.9 (156–663)	250.4±52.2 (150–332)	243.8±61.9 (113.0–403)	275.6±93.6 (113–663)	0.100
Lactate	2.1±0.2 (1.9–2.5)	1.9±0.7 (0.9–2.7)	1.9±1.1 (0.9–4.2)	1.9±0.8 (0.9–4.2)	0.539
Ferritin (ng/mL)	62.5±128.8 (5.9–405)	27.8±16.3 (5.4–51.5)	53.9±97.3 (5.3–405.4)	49.8±93.9 (5.3–405.4)	0.908
Fibrinogen (mg/mL)	267.5±93.9 (109–413)	287.8±54.6 (226–374)	251.8±51.1 (163–367)	265.3±67.0 (109–413)	0.710
D-Dimer (μg/l)	5620.9±12936.5 (192–48000)	740.4±983 (266–3280)	480.3±853.3 (84–3620)	2300.5±7789.7 (84–48000)	0.476
CK-MB (U/L)	3.2±1.7 (2.0–6.8)	2.8±1.0 (2.0–4.9)	2.5±1.3 (2.0–6.4)	2.9±1.4 (2.0–6.8)	0.703
CK (U/L)	114.3±78.5 (36–321)	16.7±39.1 (27–133)	69.3±25.7 (31–114)	85.6±53.7 (27––321)	0.288
Folate (pg/mL)	12.1±6.1 (5.2–21.8)	10.2±2.9 (5.9–13.4)	7.4±2.4 (3.8–12.9)	9.1±4.1 (3.8–21.8)	0.053
Vitamin B12 (pg/mL)	337.5±197.8 (137–709)	208.4±59.6 (141–319)	195.1±63.2 (106–290)	231.5±120.3 (106–709)	0.087
CRP (mg/dL)	0.81±0.9 (0.2–2.3)	1.3±1.6 (0.2–5.2)	1±0.85 (0.2–2.4)	1±1 (0.2–5.2)	0.667
PK (ng/mL)	0.13±0.45	0.22±0.21	0.11±0.01	0.14±0.09	0.774
Troponin I (ug/L)	0.07±0.042	0.03±0.04	0.04±0.04	0.05±0.04	0.11

ALT: Alanine aminotransferase; AST: Aspartate aminotransferase; ALP: Alkaline phosphatase; Aptt: Activated partial thromboplastin time; PT: Prothrombin time; INR: International normalized ratio; LDH: Lactate dehydrogenase; WBC: White blood cell count; Hb: Hemoglobin level; PLT: Platelet; CK-MB: Creatine kinase myocardial band; CK: Creatine kinase; CRP: C-reactive protein; PK: Procalcitonin; Min: Minimum; Max: Maximum.

The mean time from admission to negative rRT-PCR test was 7.82±1.5 (4–11) days, and all patients were discharged after testing negative on rRT-PCR tests. Association between the time to negative rRT-PCR test and age groups was examined. The mean time to a negative rRT-PCR test was 8.33±1.49 (6–11) days in the 1–71 months age group, 7.66±1.65 (6–11) days in the 72–143 months age group and 7.43±1.63 (4–10) days in the 144–216 months age group. Inter-group comparisons did not show a statistically significant difference in the meantime to a negative test among the groups (p=0.287).

HQ therapy was given as part of combination treatment to 15 symptomatic patients older than 72 months of age (72–143 months of age: four patient, 144–216 months of age: 11 patients). The mean time to a rRT-PCR negative test was 7.2±1.69 (4–10) days for the group receiving an HQ treatment protocol and 8.2±1.44 (6–11) days for the group receiving a non-HQ treatment protocol with no significant difference between the groups (p=0.054).

None of the patients had other microorganisms as demonstrated by additional tests for viral and bacterial pathogens causing pneumonia.

## Discussion

Our review of current literature showed that children had a better clinical course and lower mortality in comparison to adults in all studies on COVID-19 infections [6, 10]. This favorable clinical picture observed among children was suggested to result from differences in the pathogenesis between pediatric and adult patients, and it was proposed that high-risk groups should be defined for children [11, 12].

Consistent with the literature, children had a quite good clinical course and none of our patients had to be admitted to the pediatric intensive care unit or required mechanical ventilation in our study. Furthermore, none of the study patients had comorbidities or underlying disorders. We were lucky not to have complicated cases and probably as a result of this, we had no case fatality in contrast to mortality reports in the literature [13].

All of our patients had signs and symptoms of COVID-19 as well as a history of contact with adults who tested positive on COVID-19 rRT-PCR test. The prominent symptoms of our patients were cough (47.5%), fever (20%), weakness (35%), loss of taste sensation (5%), loss of sense of smell (5%), diarrhea (2.5%), and vomiting (2.5%). Fever was the most common symptom in studies by Wang et al. [14] and Jiehao et al. [15]; it was cough in our study, similar to the study by Xia et al. [16] However in our study symptom frequencies, there were lower than those reported in the literature [17, 18].

In the current study, we divided the patients into groups based on age and compared laboratory parameters among age groups. These comparisons showed statistically significant differences among the groups in terms of WBC, Hb, lymphocyte and PLT, AST, ALP, Aptt, Vitamin B12, folate (p<0.05). This indicates that it may not be right to classify the pediatric patients as a single age group from 1 to 18 years of age and that we should consider dividing them into several age subgroups.

Regarding laboratory parameters reported in literature, studies in adults with COVID-19 showed that lymphopenia occurred commonly and was identified as a risk factor that is associated with poor prognosis. While we are aware of the fact that, as with other parameters, lymphocytopenia in children should also be examined among different age groups, lymphopenia was detected in 8 (20%) patients, low WBC in 3 (7.5%) patients, and thrombocytopenia in 1 (2.5%) patient when all age groups were examined collectively. Much higher rates were observed in an adult study conducted by Guan et al. [19].

The “mean length of hospital stay” and “the time to rRT-PCR test negativity” were used to refer to the same outcome in the present study since all patients were discharged immediately after becoming negative on rRT-PCR test, and the mean time a negative test was 7.82±1.5 (4–11) days. The mean time to a negative test was 12 days (6–21) in Jiehao et al.’s [15] study. The reason for such a short time period observed in our study is that none of our patients required intensive care.

In addition, we compared the time to discharge or rRT-PCR test negativity time among age groups (Groups 1, 2, and 3) to see whether it was associated with age and no significant association was found. This suggests that age is not an important determinant of the time to test negativity among pediatric patients in COVID-19 infection.

At present, there are no drugs or therapeutics with proven efficacy to treat COVID-19 infection. We generated individual treatment protocols appropriate for age, weight, signs, and symptoms for our 40 hospitalized patients which consisted of azithromycin (10 mg/kg/dose in children 1–5 months, in children older than 6 months and adolescents 10 mg/kg once daily on the 1^st^ day, followed by 5 mg/kg once daily for 2–5 days.), oseltamivir (Term babies 0–12 months 3 mg/kg/dose twice a day, <15 kg children 30 mg twice a day, 45 mg twice a day for those between 15 kg and 23 kg, 60 mg twice a day for those between 23 kg and 40 kg, 75 mg twice a day for >40 kg, 5 days in total) and HQ (6.5 mg/kg/dose twice a day on the first day, followed by 3.25 mg/kg/dose twice a day for 2–5 days). Other 39 patients who did not need to be treated and monitored at hospital were followed without receiving any treatment and excluded from the study (Fig. 1). Some patients who were 72 months of age or older received combination treatment including azithromycin, oseltamivir and HQ. Subsequently, intra-group comparisons were done for treated groups to find out the effect of treatment on the time to a negative test but no significant treatment-related difference was observed among the groups.
